# Polystyrene Microplastics Can Aggravate the Damage of the Intestinal Microenvironment Caused by Okadaic Acid: A Prevalent Algal Toxin

**DOI:** 10.3390/md23030129

**Published:** 2025-03-17

**Authors:** Hong-Jia Huang, Yang Liu, Da-Wei Li, Xiang Wang, Nai-Xian Feng, Hong-Ye Li, Ce-Hui Mo, Wei-Dong Yang

**Affiliations:** Key Laboratory of Aquatic Eutrophication and Control of Harmful Algal Blooms of Guangdong Higher Education Institute, College of Life Science and Technology, Jinan University, Guangzhou 510632, China; hjhuang@jnu.edu.cn (H.-J.H.);

**Keywords:** microplastic, okadaic acid, intestinal barrier, Caco-2 cell, gut microbiota

## Abstract

As emerging contaminants, microplastics (MPs) may pose a threat to human health. Their co-exposure with the widespread phycotoxin okadaic acid (OA), a marine toxin known to cause gastrointestinal toxicity, may exacerbate health risk and raise public safety concern. In this study, the toxicity mechanisms of MPs and OA on intestinal microenvironment was explored using human Caco-2 cells as the model, which was combined with an in vitro fecal fermentation experiment. Our results showed that co-exposure to MPs (80 μg/mL) and OA (20 ng/mL) significantly decreased cell viability, increased intracellular reactive oxygen species (ROS) production, elevated lactate dehydrogenase release, impaired ABC transporter activity, promoted OA accumulation, and triggered inflammatory response compared to the control, MPs, and OA groups, indicating that co-exposure directly compromises intestinal epithelial integrity. In vitro fermentation experiments revealed that co-exposure disrupted gut microbial composition, decreasing the relative abundance of some bacteria, such as *Parasutterella* and *Adlercreutzia*, while increasing opportunistic pathogens, such as *Escherichia-Shigella*, increased. These findings provide new insights into the impact and underlying mechanisms of MPs and OA co-exposure on intestinal homeostasis, highlighting the potential health risks associated with MPs.

## 1. Introduction

Owing to their unique properties, plastics are widely utilized in daily life, resulting in serious environmental pollution, and they have been one of the largest threats to marine ecological safety [1]. Microplastics (MPs) are plastic fragments and particles typically less than 5 mm in diameter, while particles smaller than 100 nm are classified as nanoplastics (NPs) [2,3]. The ecotoxicological effects of MPs on marine organisms, including phytoplankton, zooplankton, invertebrates, and plants, have been well documented [4,5,6]. In addition, concerns about the potential risks of MPs to human health are growing and dominant.

Okadaic acid (OA), a major algal toxin ubiquitously distributed in offshore and coastal ecosystems, is the primary etiological agent of diarrheal shellfish poisoning (DSP) [7]. This marine biotoxin accumulates preferentially in filter-feeding bivalve mollusks that consume some toxin-producing dinoflagellate species, including *Dinophysis* sp. and *Prorocentrum* sp., and they also pose a considerable health risk through the food chain [8,9]. The symptoms of OA poisoning are similar to those of gastrointestinal diseases [10], and they typically appear within a few hours after ingestion [11]. Due the non-specific nature of OA’s toxic symptoms, it is often difficult to diagnose and, as a result, DSP events are frequently overlooked, potentially leading to an underestimation of their true incidence [11]. It is currently believed that the acute toxicological effect of OA is closely associated with its inhibition of serine/threonine protein phosphatases 1 (PP1) and 2A (PP2A) [12]. Of concern, OA has been identified as a potential carcinogen [13]. Cordier et al. (2000) found a significant correlation between DSP toxins exposure and increased risk of cancer, especially gastric and colon cancers [14]. Similarly, López-Rodas et al. (2006) and Manerio et al. (2008) both highlighted a significant link between shellfish consumption, especially those contaminated with DSP toxins, and an increased risk of colorectal cancer [15,16]. These studies highlight that OA not only poses a substantial environmental threat, but also has a profound and lasting impact on public health.

The co-occurrence of OA and MPs in marine environments has emerged as a critical ecological and public health concern [17,18]. These contaminants bioaccumulate in commercially important seafood species, including mussels, shrimp, and fish, posing significant dietary exposure risks to consumers [8,19,20]. The growing risks associated with concurrent exposure to these pollutants have elevated the issue to a significant global public health concern [19,20]. As a result, research is increasingly targeting the toxic impact of MPs and OA on humans, especially the effects on the gastrointestinal system [8,19,20]. The gastrointestinal system is highly specialized and complex, not only facilitating nutrient absorption [21,22], but also acting as a barrier to maintain intestinal balance and preventing the entry of pathogens and toxins [23,24]. Disruption of this barrier may result in intestinal leakage, potentially leading to serious physiological diseases [25,26], which are manifested by intestinal epithelial injury, increased permeability, and reduced mucus secretion [27,28]. Mounting evidence also highlights the gut microbiota’s essential role in maintaining gastrointestinal homeostasis [29,30,31], with environmental contaminants potentially altering microbial communities and exacerbating epithelial dysfunction [32,33]. In our earlier research, we found that simultaneous exposure to MPs and OA increased the accumulation of OA and led to severe intestinal damage in mice [8]. However, the mechanism by which combined MPs and OA exposure causes intestinal damage is unclear, whether it is caused directly by MPs and OA or indirectly through changes in the gut microbiota triggered by exposure.

As an intestinal cell model, Caco-2 has been widely used for investigating the impact of drugs and contaminants on the gut due to its morphological and functional similarities to human enterocytes [34,35]. In this study, we utilized Caco-2 monolayers to investigate the cytotoxic effects of MPs and OA, with the aim of elucidating the underlying mechanisms of intestinal damage induced by their combined exposure. To this end, we employed a multi-parametric approach to evaluate intestinal barrier dysfunction, including assessments of cell viability, oxidative stress biomarkers, lactate dehydrogenase (LDH) release, ABC transporter activity, and intracellular OA accumulation. Additionally, we used 16S rRNA sequencing to characterize microbial community shifts in an in vitro fermentation system. Our findings provide further evidence for understanding the combined effects of MPs and OA on the intestinal tract and to raise awareness about the risks of MPs pollution.

## 2. Results

### 2.1. Effects of MPs and OA on the Viability of Caco-2 Cells

**Figure 1:**
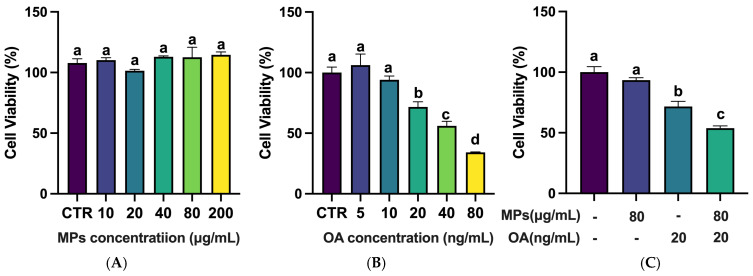
Cytotoxicity induced by MPs and/or OA (*n* = 3). (**A**) Cell viability of MPs on Caco-2 cells. (**B**) Cell viability of OA on Caco-2 cells. (**C**) Cell viability of co-exposure to MPs and OA on Caco-2 cells. All data represent the mean ± SEM. Bars with different letters (a,b,c,d) are significantly different (*p* < 0.05).

As shown in Figure 1A, 5 μm MPs with concentrations ranging from 10 μg/mL to 200 μg/mL had little effect on Caco-2 cell viability, with no notable differences between the MPs and control group. However, the OA demonstrated a dose-dependent effect on Caco-2 cell viability. While 5 and 10 ng/mL OA did not affect cell viability, 20, 40, and 80 ng/mL OA significantly reduced cell viability (Figure 1B). The half maximal inhibitory concentration (IC50) of OA on Caco-2 cells was 18.12 ng/mL. Therefore, combined with previous studies [36], 80 μg/mL MPs and 20 ng/mL OA were selected for a further combined exposure experiment. As illustrated in Figure 1C, the cell viability of the co-exposure group exhibited a significantly decreased trend (*p* < 0.05) compared to the control, MPs, and OA groups, indicating a potential synergistic effect of the MPs and OA on Caco-2 viability.

### 2.2. Co-Exposure of MPs- and OA-Enhanced ROS Production and Triggered Inflammatory Responses

**Figure 2:**
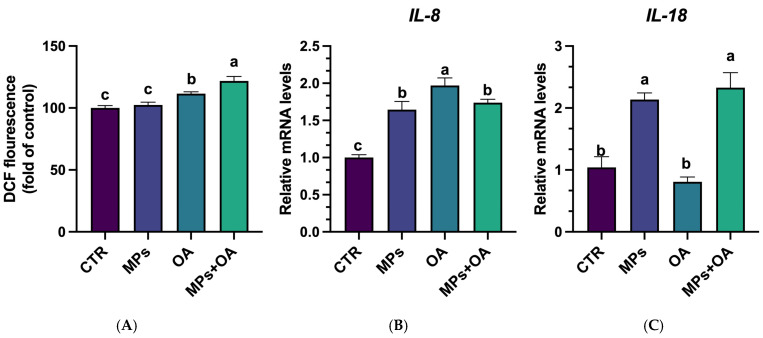
Effect of the MPs (80 μg/mL) and OA (20 ng/mL) on the ROS production and inflammatory responses in Caco-2 cells. (**A**) Intracellular ROS content, *n* = 3. (**B**) IL-8 mRNA level, *n* = 4. (**C**) IL-18 mRNA level, *n* = 4. All data represent the mean ± SEM. Bars with different letters (a,b,c) are significantly different (*p* < 0.05).

Compared with the control group, there was no significant change in the intracellular ROS levels in the MPs group (Figure 2A). However, the ROS levels in the OA and co-exposure groups were significantly higher than that in the control group (*p* < 0.05), and the ROS levels in the co-exposure group were significantly higher than that in the OA group (*p* < 0.05). These outcomes suggest that co-exposure to MPs and OA induces Caco-2 cells to produce more ROS.

Compared with the control, the expression of *IL-8* was significantly increased in the MPs, OA, and co-exposure groups (*p* < 0.05), but there was no significant difference in the *IL-8* expression between the co-exposure group and the groups exposed to MPs or OA (Figure 2B). *IL-18* expression was significantly increased in the MPs and co-exposure groups compared to the control (*p* < 0.05), with no notable change in the OA group. Additionally, the *IL-18* mRNA level in the co-exposure group was significantly higher than those in the OA group (*p* < 0.05), but no significant difference was observed between the MPs and co-exposure groups. These findings indicate that combined exposure to MPs and OA can trigger inflammatory responses in Caco-2 cells.

### 2.3. MPs Promoted the Intracellular Accumulation of OA in Caco-2 Cells

**Figure 3:**
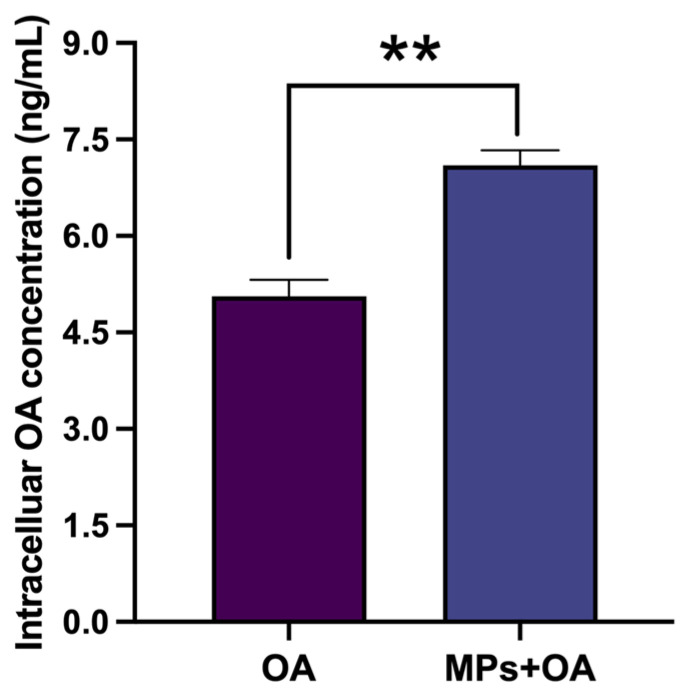
The intracellular OA concentration in Caco-2 cells (*n* = 6). All data represent the mean ± SEM. ** represent *p* ˂ 0.01 between the OA group (20 ng/mL) and co-exposure group (MPs 80 µg/mL + OA 20 ng/mL).

The intracellular concentration of OA in Caco-2 cells was significantly higher in the co-exposure group (7.10 ± 0.52 ng/mL) compared to the OA group (5.06 ± 0.57 ng/mL) (*p* < 0.01) (Figure 3).

### 2.4. Changes in the LDH Release and ABC Transporter Activity

**Figure 4:**
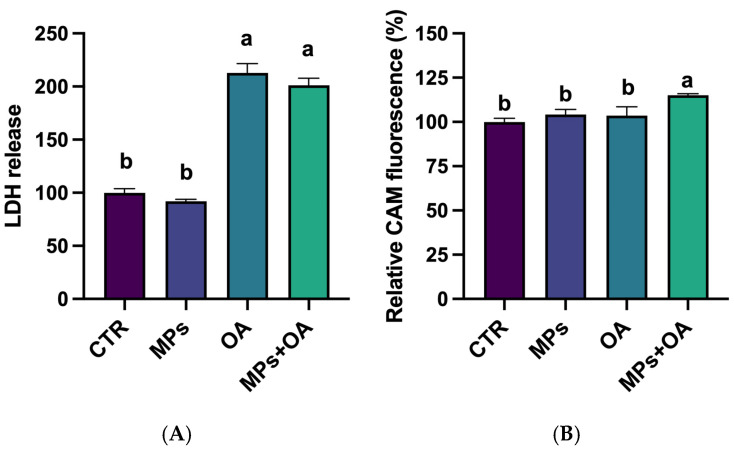
The effects of MPs (80 μg/mL) and OA (20 ng/mL) on the LDH release and ABC transporter of Caco-2 cells (*n* = 3). (**A**) LDH release from Caco-2 cells. (**B**) CAM accumulation in Caco-2 cells. All data represent the mean ± SEM. Bars with different letters (a,b) are significantly different (*p* < 0.05).

Figure 4 shows the changes in LDH release and ABC transporter activity. Exposure to MPs alone did not significantly alter LDH release compared to the control (*p* > 0.05). In contrast, both the OA exposure and combined exposure groups caused a significant increase in LDH release (*p* < 0.05), with OA and co-exposure groups showing 2.1-fold and 2.0-fold increases, respectively, compared to the control group. However, no significant difference was observed between the combined exposure group and the OA group. These results indicate that the MPs had no effect on the LDH release in Caco-2 cells, and the increased LDH release observed after co-exposure to MPs and OA may be attributed to the role of OA.

As illustrated in Figure 4B, neither MPs nor OA exposure alone resulted in significant changes in CAM accumulation in the cells compared to the controls. In contrast, co-exposure to MPs and OA significantly increased CAM accumulation compared with the control, MPs, and OA groups. These findings indicate that concurrent exposure to MPs and OA synergistically attenuates ABC transporter-mediated efflux, potentially enhancing intracellular toxin accumulation.

### 2.5. Effects of the MPs and OA Exposure on the Expression of Genes Related to the Intestinal Barrier

**Figure 5:**
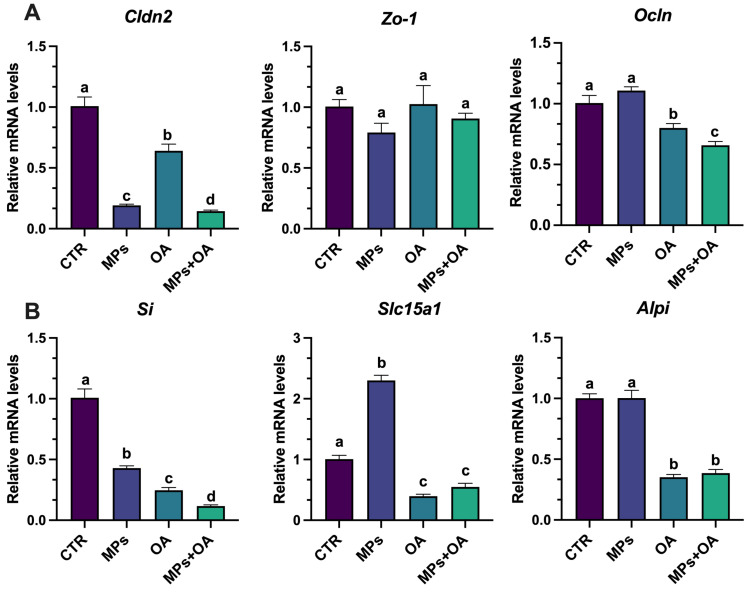
The effects of MPs (80 μg/mL) and OA (20 ng/mL) on the expression of genes associated with the intestinal barrier in Caco-2 cells (*n* = 4). (**A**) The genes related to tight junction components. (**B**) The genes related to brush border enzymes. All data represent the mean ± SEM. Bars with different letters (a,b,c,d) are significantly different (*p* < 0.05).

Changes in the expression of genes related to intestinal epithelium function and morphology were assessed using qPCR. Six genes were selected, three associated with the tight junction components (*claudin-2* [*Cldn2*], *occludin* [*Ocln*], and *zonula occludens-1* [*Zo-1*]) and three associated with the brush border function (*alkaline phosphatase* [*Alpi*], *sucrase-isomaltase* [*Si*], and *solute carrier family 15 member 1* [*Slc15a1*]). As illustrated in Figure 5A, the expression of *Cldn2* in the MPs, OA, and co-exposure groups was significantly decreased compared to the control group (*p* < 0.05), and the expression of *Cldn2* in the co-exposure group was lower than that in the MPs and OA groups (*p* < 0.05). *Zo-1* expression remained unchanged across all groups, showing no significant differences from the control. In contrast, *Ocln* expression was notably down-regulated (*p* < 0.05) in the OA and co-exposure groups, with the co-exposure group displaying a greater reduction than the MPs and OA groups (*p* < 0.05). No significant changes in *Ocln* were observed in the MPs group.

As shown in Figure 5B, after 24 h of exposure to MPs and OA, the *Si* expression in the MPs, OA, and co-exposure groups was significantly lower compared to the control group (*p <* 0.05), while the *Si* expression in the co-exposure group was significantly lower than that in the MPs and OA groups (*p* < 0.05). Compared with the control group, *Slc15a1* expression was significantly up-regulated in the MPs exposure group, but it was significantly decreased in the OA and co-exposure groups (*p <* 0.05). *Alpi* expression was notably reduced in both the OA and co-exposure groups compared to the control group, with no significant difference between these two groups. There was no significant change found in *Alpi* expression in the MPs group. These findings indicate that the co-exposure to MPs and OA has a significant impact on intestinal barrier function.

### 2.6. The Effect of MPs and OA Exposure on Gut Microbiota

**Figure 6:**
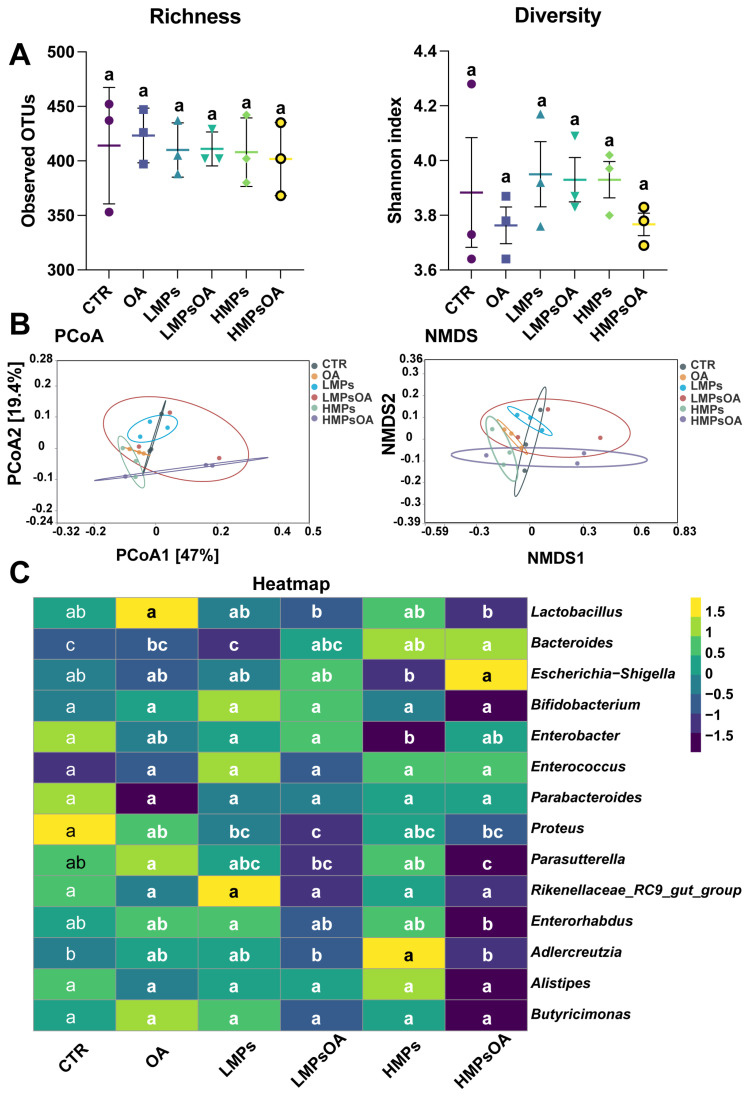
The effects of MPs and OA on gut microbiota (*n* = 3). (**A**) Richness and diversity analysis. (**B**) Bacterial β-diversity revealed by NMDS and PCoA analysis. (**C**) The relative abundance of bacteria was visualized as the mean value within the corresponding group after homogenization by Z-score. a, b, c, ab, bc and abc are statistical results of difference between groups, and groups with same letter represents no significant difference between them (*p* > 0.05). LMPs, 100 μg/mL MPs; HMPs, 1 mg/mL MPs. All data represent the mean ± SEM.

The impact of MPs and/or OA on bacterial alpha and beta diversity were assessed after 24 h using 16S rRNA high-throughput sequencing. Based on the observed OTUs and Shannon indexes, there were no significant differences in richness and diversity between the various treatments (i.e., the control, OA, low concentration of MPs (LMPs), high concentration of HMPs, LMPs + OA, and HMPs + OA treatments). These findings suggest that short-term exposure to MPs and OA has a limited effect on the diversity of the gut microbiota in mice (Figure 6A).

In terms of principal coordinate analysis (PCoA) and nonmetric multidimensional scaling (NMDS) analysis, exposure to MPs and OA led to varying degrees of bacterial community dispersion. Significant differences were observed between the LMPs, HMPs, co-exposure, and control groups. The HMPs + OA group was clearly distinct from both the control and OA groups, as demonstrated by PCoA (Figure 6B). Both variance using distance matrices (Adonis) and molecular variance (Amova) tests gave similar results (Table S3).

At the phylum level, no significant differences in the gut microbiota were observed after MPs and OA exposure compared to the control group (Figure S2A). Firmicutes and Bacteroidetes were the two dominant phyla. Compared to the control group, MPs group (LMPs and HMPs), and OA group, the relative abundances of Firmicutes and Actinobacteria in the combined exposure group (LMPs + OA and HMPs + OA) showed a downward trend.

After exposure to MPs and OA, the relative abundance of 14 bacterial genera changed to varying degrees (Figure S2B), with some genera showing a dose-dependent response (Figure 6C). In comparison to the control group, the HMPs + OA group exhibited a marked reduction in the relative abundance of *Proteus* and *Parasutterella*, whereas *Bacteroides* levels significantly increased. Additionally, the relative abundance of *Escherichia_Shigella* exhibited an upward trend, while *Lactobacillus*, *Rikenellaceae_RC9_gut_group*, *Enterorhabdus*, *Adlercreutzia*, *Alistipes*, and *Butyricimonas* showed a downward trend.

## 3. Discussion

The gastrointestinal (GI) tract is a complex dynamic system composed of various biological components, including epithelial cells, multiple cell types, luminal contents, microbiota, and the extracellular matrix [37,38]. This intricate system serves as the primary barrier against xenobiotic invasion, maintaining systemic homeostasis by regulating mucosal permeability [39]. As the first site of contact for orally ingested contaminants, the intestinal epithelium is particularly vulnerable to toxic insults [40,41,42]. Intestinal barrier dysfunction is intimately associated with perturbations in epithelial integrity and microbiota–host interactions [27]. In our previous study, we found that combined exposure to MPs and OA damaged the integrity of intestinal barrier in mice [8], but it was not clear whether this damage was caused directly by MPs and OA, or indirectly through changes in gut microbiota triggered by exposure. In this study, Caco-2 cells were used as a model, and the mechanism of co-exposure to MPs and OA on intestinal barrier was investigated combined with in vitro fermentation experiments.

We found that MPs had no obvious cytotoxicity to Caco-2 cells, but co-exposure of MPs (80 μg/mL) and OA (20 ng/mL) notably enhanced the cytotoxicity of OA to Caco-2 cells, demonstrating a synergistic toxic effect. This finding aligns with previous reports [20,36], which have highlighted that co-exposure to MPs and other environmental pollutants often exacerbates their combined toxicity and promotes bioaccumulation in biological systems [43,44,45]. ROS are chemically reactive molecules that are produced naturally during oxygen metabolism and play a critical role in cellular stress responses [46,47]. Elevated ROS levels are known to cause cellular damage [48], and their accumulation can be triggered by factors such as UV radiation and toxicants [46]. Notably, our analysis revealed that MPs/OA co-exposure led to substantial increases in intracellular ROS production, and it also up-regulated the expression of pro-inflammatory cytokines *IL-8* and *IL-18* in Caco-2 cells, suggesting that simultaneous exposure to MPs and OA enhances ROS production and triggers inflammatory responses in Caco-2 cells.

Lin et al. (2021) demonstrated that nanoplastics (NPs) exacerbate the toxicity and intracellular accumulation of arsenic in AGS cells by inhibiting ABC transporter activity [19]. Similarly, other studies have shown that both 5 μm and 100 nm PS-MPs can inhibit the activity of the ABC transporter [36]. Our LC-MS/MS analysis revealed that the intracellular content of OA was significantly higher in the co-exposure group than in the OA group, and the ABC transporter activity was also significantly reduced, which may have contributed to the increased cytotoxicity caused by the combined exposure [36,44]. ABC transporters are essential for the absorption, distribution, and excretion of drugs and toxins in the small intestine, liver, and kidneys [49], and their functional activity is tightly linked to plasma membrane integrity [50,51]. We evaluated it using lactate dehydrogenase (LDH) release assays [52], which showed that exposure to MPs alone did not affect LDH release in Caco-2 cells; however, the OA group and the combined exposure group (MPs and OA) exhibited a markedly increase in LDH release, further suggesting that combined exposure can damage cell membrane integrity.

To characterize the impact of combined MP and OA exposure on intestinal barrier function, we analyzed the expression changes in key barrier-related genes. Zhao et al. (2021) reported that imidacloprid disrupted the intestinal barrier of Caco-2 cells by significantly down-regulating tight junction genes, including *Olcn*, *Zo-1*, and *Claudin-1*, highlighting its impact on barrier permeability [53]. Similarly, our study found that combined exposure to MPs and OA significantly down-regulated intestinal barrier-related genes like *Cldn2* and *Olcn* in Caco-2 cells, suggesting adverse effects on barrier permeability. García-Rodríguez et al. (2018) investigated the effects of different shaped nano-TiO_2_ particles on the intestinal barrier in a Caco-2/HT29 co-culture model, and they found that these particles affected the expression of some crucial genes, such as *Alpi*, *Si*, and *Slc15a1*, thereby compromising barrier integrity [54]. *Alpi*, a protein located on the brush border of the intestinal mucosa, is crucial for maintaining the homeostasis of the intestine [55]. Both *Si* and *Slc15a1* are also important markers for evaluating intestinal barrier dysfunction [56]. When the combined biological or toxic effects of two chemicals are equal to or greater than the effects of each chemical alone, it indicates additive or synergistic interactions [57,58]. The gene expression patterns of *Si, Slc15a1*, and *Alpi* in the co-exposure group suggest that the combined exposure to MPs and OA may impair intestinal barrier function through additive or synergistic effects.

Gut microbiota plays a vital role in regulating host energy metabolism, secretion, and immune responses, while also contributing to the pathogenesis of multiple diseases [29,59]. Environmental pollutants can disrupt the composition of gut microbiota, leading to intestinal epithelium dysfunction and serious consequences [33]. Liu et al. (2020, 2022) suggested that OA can disrupt the intestinal epithelial microenvironment, lead to the imbalance of gut microbiota, and it can directly affect the abundance of some bacterial genera [9,10]. Huang et al. (2021) investigated the impact of tetrabromobisphenol A (TBBPA) and PE-MPs on bacterial diversity and structure, revealing the inhibition of beneficial bacteria and the proliferation of opportunistic bacteria [46]. Similarly, our study demonstrated that co-exposure to MPs and OA led to alterations to the bacterial community to varying degrees. The relative abundances of some bacteria genera, such as *Parasutterella* and *Enterorhabdus*, were reduced, while *Bacteroides* and *Escherichia-Shigella* showed increases. These microbial shifts are associated with perturbations in host physiological processes [29,60,61]. For instance, *Parasutterella* is a key genus maintaining a healthy gut microbiota [62]. Although its abundance has been linked to the development of Irritable Bowel Syndrome, studies in animal models have shown that high-fat diets (HFD) can significantly reduce the *Parasutterella* level [63]. Notably, an increase in *Parasutterella* abundance has been associated with reduced serum lipid profile [64]. In addition, a relatively high abundance of *Escherichia-Shigella* was found in the fecal microbiota of Crohn’s disease patients [65]. Similar changes were observed in a model of *Escherichia coli* O101-induced diarrhea in rats [66]. Though the impact on host health cannot be simply determined by changes in the relative abundance of certain bacterial genera, the microbial dysbiosis caused by pollutants, especially those regarding a decrease in beneficial bacteria and an increase in opportunistic pathogens, may disrupt the dynamic balance of the gut microbiota. The effect of MPs and OA co-exposure on gut microbiota may also be an important reason for their combined toxicity, which requires further investigation. Notably, it is important to acknowledge that certain gut microbes may not survive in vitro fermentation systems, and the microbiota profiles in feces may not fully recapitulate those in the intestinal lumen. These factors introduce limitations in interpreting the full impact of MPs/OA co-exposure on gut microbiota. However, shifts in specific microbial populations suggest that combined exposure causes perturbations of the gut microbiota, which, together with the direct effects of toxins, lead to increased damage to the gut barrier.

## 4. Materials and Methods

### 4.1. MPs and OA

Polystyrene microplastics (PS-MPs; 5.0 μm in diameter) were sourced from Tianjin Base Line Chromatography Technology Development Center and characterized using established protocols. They were suspended in phosphate-buffered saline (PBS) at 1 mg/mL [8]. Before cell exposure, MPs were diluted in a fetal bovine serum (FBS)-containing culture medium. Additional characterization details are provided in Figure S1 and Table S1.

OA (HPLC purity ≥ 95%) was obtained from the Algal Science Inc. (Taibei, Taiwan, China). A stock solution was prepared by dissolving OA in 100 μL of dimethyl sulfoxide (DMSO), which was then diluted in PBS to a final concentration of 100 μg/mL [8].

### 4.2. Cell Treatment Protocol

The Caco-2 cells (Procell, Wuhan, China) were cultured in an RPMI-1640 medium (Gibco, Thermo Fisher Scientific, Waltham, MA, USA) supplemented with 10% (*v*/*v*) FBS (Lonsera, Uruguay) and 1% penicillin-streptomycin solution, which was then maintained in a 37 °C incubator (Thermo Fisher Scientific, Waltham, MA, USA). Cells at 80–90% confluence were treated with MPs and/or OA for 24 h. Stock solutions of MPs and OA were diluted with RPMI-1640 to final concentrations (0, 10, 20, 40, 80, and 200 μg/mL MPs; 0, 5, 10, 20, 40, and 80 ng/mL OA). Based on the results, the MPs at 80 μg/mL were combined with OA at 20 ng/mL for further cytotoxicity evaluation.

### 4.3. Cell Vitality Assays

Cell viability was evaluated using a CCK-8 assay kit (Biosharp, Hefei, China). After treatment with MPs and/or OA, the cells were incubated with a CCK-8 solution at incubator for 1 h. Absorbance (450 nm) was then measured with a Bio-TEK microplate reader (Cytation5, BioTek Instruments, Winooski, VT, USA) for assessing cell viability. Cell viability was calculated using the following formula:

Cell viability (%) = [(C_e_ − C_0_)/(C_c_ − C_0_)] × 100%,

where C_e_ and C_c_ represent the absorbance of the experimental and control groups, respectively, and C_0_ is the absorbance of the culture medium.

### 4.4. Quantitative Determination of OA

OA concentration was measured using LC-MS/MS, as described in our previous study [8]. Briefly, the cell culture was centrifuged at 200× *g* for 3 min, washed three times with PBS, and homogenized in 1 mL of 100% methanol. The extract was then evaporated under nitrogen, and the resulting supernatant was filtered through a 0.22 μm membrane filter. OA levels were quantified using a standard curve.

### 4.5. Measurement of Intracellular ROS

The ROS levels in Caco-2 cells were assessed using a ROS assay kit (Beyotime, Shanghai, China) after exposure to MPs or/and OA, in accordance with a previous study [67]. The cells were treated for 24 h, washed with PBS, and then incubated with 20 nM of Dichloro-dihydro-fluorescein diacetate (DCFH-DA) at 37 °C for 30 min in the dark. Intracellular ROS levels were then measured using a multi-mode microplate reader (CLARlOstar, BMG LABTECH, Ortenberg, Germany) at 488 nm excitation and 525 nm emission.

### 4.6. Evaluation of Lactate Dehydrogenase Release

LDH levels were assessed with a LDH assay kit (Beyotime, China) after 24 h of exposure. The supernatant was obtained by centrifugation (200× *g*), followed by the addition of 60 μL of LDH reagent. After 30 min incubation at room temperature in the dark, the absorbance (490 nm) was recorded using a multi-mode microplate reader (CLARlOstar, BMG LABTECH, Ortenberg, Germany).

### 4.7. Determination of the Transmembrane ABC Transporter Activity

The transmembrane ABC transporter activity was determined using Calcein-AM (CAM, Beyotime, China), in accordance with the protocol outlined by Lin et al. (2021) [44]. Following 24 h of exposure to MPs and/or OA, cells were incubated with 0.25 mM of CAM for 30 min. Hoechst 33,342 staining was used for cell normalization. CAM fluorescence was measured using a multi-mode microplate reader (CLARlOstar, BMG LABTECH, Ortenberg, Germany) at 494/516 nm, while Hoechst 33,342 was detected at 350/461 nm.

### 4.8. RT-qPCR Analysis

RNA was extracted with TRIzol reagent, and its purity was assessed by measuring the A260/280 and A260/230 ratio. cDNA synthesis and RT-qPCR were carried out using the reverse transcription kit (R233–01) and qPCR kit (Vazyme, Nanjing, China). PCR cycling conditions were set at 95 °C for 30 s, followed by 40 cycles of 95 °C for 10 s and 60 °C for 30 s. The mRNA levels were normalized to 18S RNA and beta-2-microglobulin (B2m). Primers are provided in Table S2.

### 4.9. Feces Collection and Fermentation Culture

Thirty-two 8-weeks-old female C57BL/6J mice (Approval number: IACUC-20220314-09) with a 20 ± 2 g weight were housed for 10 days under controlled conditions (21~26 °C, 40~80% relative humidity, and a 12 h light/dark cycle). The fecal samples were collected without any prior treatment and mixed with sterilized modified saline solution (0.5 g/L cysteine-HCl and 9.0 g/L NaCl) to prepare a 10% (*w*/*v*) suspension. The supernatant was obtained after centrifugation (300× *g*, 4 °C, 5 min) for in vitro fermentation.

The fermentation medium was prepared as described by Liu et al. (2022) [10]. Sterile vitamin K_1_ (0.0001% final concentration) and sterile hemin (5 mg/L final concentration) were added to a sterilized GAM (Hopebiol, Qingdao, China). The collected supernatant was added into the fermentation medium in a 1: 20 ratio by volume. Considering the fact that OA at 50 nM could inhibit cell activity and that the richness and diversity of the bacterial community were not affected after 24 h exposure to 50, 100, 250, and 1000 nM of OA [10], the OA exposure concentration was set at 100 nM (equivalent to 80.5 ng/mL), and the MPs were set at concentration of 100 μg/mL and 1 mg/mL. The experiments were divided into 6 groups: (a) an equivalent solvent solution group (control); (b) low-concentration MPs group (LMPs, 100 μg/mL MPs); (c) high-concentration MPs group (HMPs, 1 mg/mL MPs); (d) OA group (80.5 ng/mL); (e) LMPs + OA group (100 μg/mL MPs and 80.5 ng/mL OA); and (f) HMPs + OA group (1 mg/mL MPs and 80.5 ng/mL OA). Culture conditions were 37 °C, with shaking at 200 rpm under anaerobic conditions for 24 h. Following centrifugation at 4000× *g* for 10 min at 4 °C, the microbiota was separated and frozen at −80 °C for further sequencing.

### 4.10. 16S rRNA Analysis of Fermentation Microbiota

DNA extraction and 16S rRNA analysis of cultured microbiota were performed following established protocols [9]. Genomic DNA (gDNA) was extracted, and its concentration and purity were assessed using a Nanodrop 2000 (Thermo Fisher Scientific, USA). The V3-V4 regions of 16S rRNA were amplified using the specific primers 338F and 806R [9]. PCR was conducted on a BioRad S1000 (Bio-Rad Laboratory, CA, USA), with an initial denaturation at 94 °C for 5 min, followed by 30 cycles of denaturation (94 °C for 30 s), annealing (52 °C for 30 s), and extension (72 °C for 30 s), concluding with a final extension at 72 °C for 10 min. PCR products were verified on a 1% agarose gel, and sequencing libraries were prepared. Paired-end sequencing (250 bp reads) was carried out on the Illumina Nova 6000 platform (Illumina, San Diego, CA, USA).

After filtering with fastp v0.14.1 [68], and removing the barcode and primer sequences with cutadapt v1.14, paired-end clean reads were merged using usearch-fastq_mergepairs v10 with default parameters. Sequences were clustered into operational taxonomic units (OTUs) at 97% similarity using UPARSE v10.0.240 [69]. Chimeric sequences were removed by aligning the OTUs to the SILVA database [70], and the OTU table was generated with USEARCH. Taxonomic classification of the representative sequences was performed using the RDP classifier [71]. Alpha diversity was assessed based on OTU richness, while beta-diversity was calculated using Bray–Curtis dissimilarity.

### 4.11. Statistical Analysis

Data are expressed as the mean ± SEM and were analyzed using R software (V4.4.2) and Graphdpad Prism 9.0 (GraphPad Prism, Inc., La Jolla, CA, USA). Statistical significance was determined using one-way ANOVA, followed by the least significant difference test for group comparisons. For data with non-homogeneous variances, Welch’s ANOVA was applied, followed by the Games–Howell method after normalization.

## 5. Conclusions

Co-exposure to MPs and OA may exacerbate intestinal barrier damage, potentially through various pathways. Our findings demonstrated that the combination of MPs and OA can disrupt membrane integrity and inhibit ABC transporter activity, leading to increased OA accumulation and heightened toxicity in Caco-2 cells. Additionally, co-exposure to MPs and OA significantly altered gut microbiota composition, reducing the abundance of beneficial bacteria like *Lactobacillus*, while promoting the growth of opportunistic pathogens like *Escherichia-Shigella*. These results suggest that co-exposure to MPs and OA can directly damage intestinal epithelial cells and disrupt microbial homeostasis. The impairment of the intestinal barrier and gut microbiota imbalance are key factors contributing to the aggravated intestinal toxicity of OA. Although this study provides valuable insights, given the variability of exposure conditions and the limited replicates, these findings will need to be strengthened in the future with more rigorous experimental designs and larger sample sizes, which could help to better understand the broader implications of MPs as environmental contaminants.

## Data Availability

The original data presented in this study are included in the article/Supplementary Material.

## Supplementary Materials

The following supporting information can be downloaded at: https://www.mdpi.com/article/10.3390/md23030129/s1, Figure S1: The SEM image of MPs; Figure S2: The relative abundance of bacteria taxa at the phylum (A) and genus (B) level after exposure of MPs and OA (*n* = 3). All data represent the mean ± SEM; Table S1: Characterization of MPs; Table S2: Primer sequences for qPCR; Table S3: Dissimilarity test between groups based on the Bray–Curtis distance (*n* = 3).
