# Supplementary material for: Polystyrene Microplastics Can Aggravate the Damage of the Intestinal Microenvironment Caused by Okadaic Acid: A Prevalent Algal Toxin
Source: Mar Drugs. 2025 Mar 17;23(3):129. doi: 10.3390/md23030129 (PMC11943709; doi:10.3390/md23030129)
Supplement: Supplementary file 1 [file marinedrugs-23-00129-s001.zip › marinedrugs-3487620-supplementary.pdf]

Supporting Information

Polystyrene microplastics could aggravate the disruption of OA on intestinal microenvironment

Hong-Jia Huang, Yang Liu, Da-Wei Li, Xiang Wang, Nai-Xian Feng, Hong-Ye Li, Ce-Hui Mo*, Wei-Dong Yang*

Key Laboratory of Aquatic Eutrophication and Control of Harmful Algal Blooms of Guangdong Higher Education Institute, College of Life Science and Technology, Jinan University, Guangzhou 510632, China.

Figure S1 The SEM image of MPs

Figure S2 The relative abundance of bacteria taxa at phylum (A) and genus (B) level after exposure of MPs and OA (n = 3). All data represent the mean ± SEM.

Table S1 Characterization of MPs

Table S2 Primer sequences for qPCR.

Table S3 Dissimilarity test between groups based on Bray-Curtis distance (n=3)

Figure S1 The SEM image of MPs

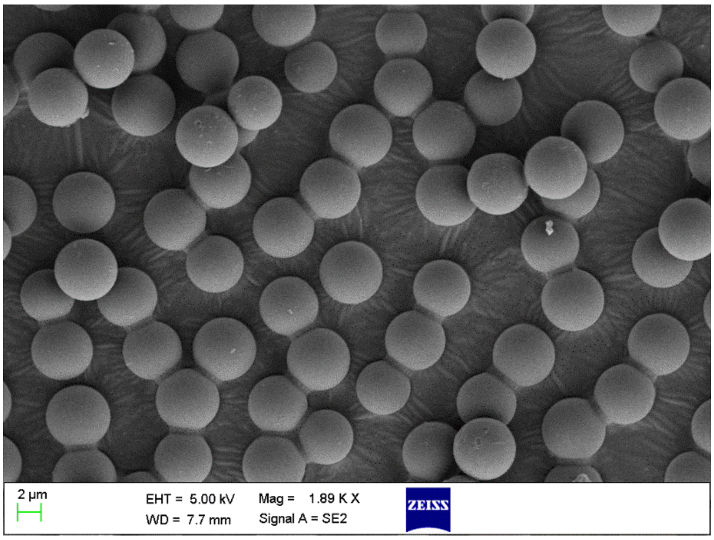

Figure S2 The relative abundance of bacteria taxa at phylum (A) and genus (B) level after exposure of MPs and OA (n = 3). All data represent the mean ± SEM.

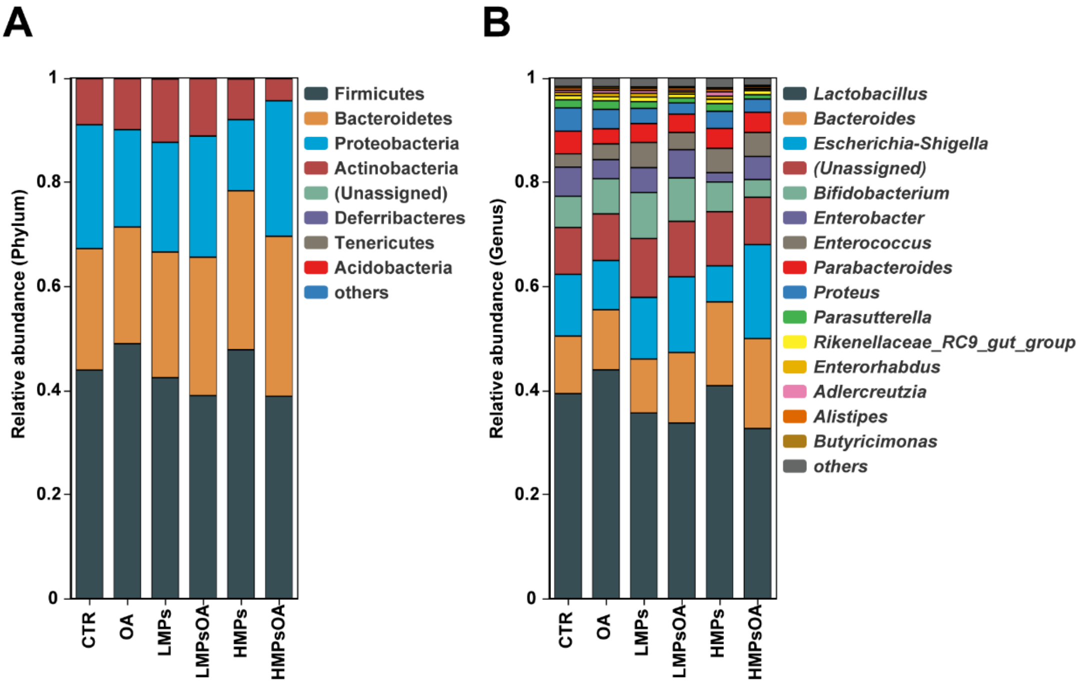

Table S1 Characterization of MPs

MPs (distributed in PBS)	
Mean	5.501 μm
Median	5.233 μm
Mode	4.982 μm
S. D	1.603 μm
C. V	29.1 %
Zeta Potential (mV)	-10.64±2.27

Table S2 Primer sequences for qPCR.

Genes	Gene description	Primer sequence (5'-3')	Products size (bp)
18S rna	ribosomal protein S18 (RPS18)	F: GATGGAAAATACAGCCAGGTCCTA<br>R: TTCTTCAGTCGCTCCAGGTCTT	74
B2m	beta-2 microglobulin	F: GGTTTCATCCATCCGACATT<br>R: GGCAGGCATACTCATCTTTTTC	156
Si	sucrase-isomaltase	F: TGGTGGCACTGTTATCCGAC<br>R: GACCACCACGGACATGTAGG	257
Slc15a1	solute carrier family 15 member 1	F: CTTCGATGCTGTGCTGTACC<br>R: GGCCAAGTGTCACCATCTCT	242
Alpi	alkaline phosphatase, intestinal	F: GTCCATCCTGTACGGCAATG<br>R: ACATGCGCTACGAAGCTCTG	216
Cldn2	claudin 2	F: TACTCACCACTGGTGCCTGA<br>R: GAGAGCTCCTTGTGGCAAGA	184
Ocln	occludin	F: ACAGACTACACAACTGGCGG<br>R: GCAGCAGCCATGTACTCTTC	236
Zo-1	tight junction protein 1	F: GAGAGGTGTTCCGTGTTGTG<br>R: GCTGCGAAGACCTCTGAATC	209
IL-8	interleukin 8	F: GAGATAATGCACCCCGGACC<br>R: TGTTCTCACAGGAGAGAGTTGA	120
IL-18	interleukin 18	F: ATCGCTTCCTCTCGCAACAA<br>R: CTTCTACTGGTTCAGCAGCCATCT	85

Table S3 Dissimilarity test between groups based on Bray-Curtis distance (n=3)

Dissimilarity test (Compared with CTR)	Adonis		Amova	
	R2	*p*-value	Fs	*p*-value
CTR vs OA	0.192	0.6	-0.00021	0.594
CTR vs LMPs	0.286	0.3	0.00299	0.299
CTR vs LMP + OA	0.284	0.2	0.00475	0.199
CTR vs HMPs	0.533	0.1	0.016	0.102
CTR vs HMP + OA	0.366	0.1	0.00987	0.101

R2 represents the interpretation degree of sample differences between groups. Fs indicates F test value.
